# The influence of user interface design on task performance and situation awareness in a 3-player diner's dilemma game

**DOI:** 10.1371/journal.pone.0230387

**Published:** 2020-03-17

**Authors:** Tingwei Jiang, Huicong Fang

**Affiliations:** School of Psychology and Cognitive Science, East China Normal University, Shanghai, China; Middlesex University, UNITED KINGDOM

## Abstract

To understand the influence of user interface on task performance and situation awareness, three levels of user interface were designed based on the three-level situation awareness model for the 3-player diner’s dilemma game. The 3-player diner's dilemma is a multiplayer version of the prisoner's dilemma, in which participants play games with two computer players and try to achieve high scores. A total of 117 participants were divided into 3 groups to participate in the experiment. Their task performance (the dining points) was recorded and their situation awareness scores were measured with the Situation Awareness Global Assessment Technique. The results showed that (1) the level-3 interface effectively improved the task performance and situation awareness scores, while the level-2 interface failed to improve them; (2) the practice effect did exist in all three conditions; and (3) the levels of user interface had no effect on the task learning process, implying that the learning rules remained consistent across different conditions.

## Introduction

With the development and popularization of network and smart devices, people have become familiar with user interfaces (UIs). In the process of human-computer interaction, the interface plays a vital role [1]. A productive and stimulating interface helps ensure high-quality interactions. Therefore, it is necessary to explain the influence of UI design on human behavior and to set standards to measure the characteristics of interfaces.

In this study, the 3-player diner’s dilemma game was employed to explore the impact of UI design on task performance and situation awareness (SA). The 3-player diner's dilemma is a multi-player version of the prisoner's dilemma, which has been widely applied in research in the economics, psychology, and political science fields [2]. Based on the SA theory and the diner's dilemma game, we hoped to explore the role of UI design in task performance and SA.

### Information availability in UI design

As the medium of human-computer interaction, people need the information provided by UIs to complete a variety of tasks. So, information display plays an important role in human-computer interaction. However, according to Lucas and Nielsen, it is difficult to design a graphical interface because there are many variables that affect the development of a computer-based information system [3]. What and how much information should be presented on the interface has long been an intriguing question.

A naïve approach would be to put as much information as possible into the UI; however, this is untenable and “less is more” has been proved to be true in many cases. In some decision studies, researchers found that presenting more information would make participants feel more confident and lead to lower accuracy in decision making [4]. Todd et al. noted that sometimes people's decisions rely on limited information [5]. The same can be found in UI research. For example, Davies et al. studied the influence of menus on task performance during command learning in a word processing application. The results showed that the group without menus performed better than the group with menus [6]. Xuan et al. used a train simulation driving game to explore the relationship between UI information and task performance, and the results also showed that more information did not mean better performance [7].

Therefore, the amount of information and how it is presented in the UI should be carefully considered. According to Tufte, “attractive displays of statistical information… display an accessible complexity of detail” [8]. In other words, a good information display should contain task-relevant information and be presented in a reasonable way that does not overwhelm the users. This sentiment was echoed by human factors psychologists such as Sweller [9,10] and was a component of the ISO standards for interface design.

Under the guidance of these principles, the results still depended on the circumstances. Laura et al. studied information availability in a simulated command and control environment. The results indicated that increasing the volume of information, even when it was accurate and task-relevant, was not necessarily beneficial to decision-making performance [11]. Davidsson and Alm's research on driving information showed that drivers' need for information was very complex. People usually need different information in different contexts [12]. Therefore, we argue that the design of a UI should reflect human cognitive processes and fully consider cognitive processing limits and capabilities. There is some evidence to support this assumption, such as the study by Dina et al., which found that when users could customize their UIs, errors were reduced and user acceptance was improved [13]. In summary, to achieve a well-designed UI, we should not only study the task itself, but also take a deep look at the cognitive processes of the human operators.

### SA and SAGAT

One theory that facilitates the understanding of humans’ cognitive processes and has been effectively applied to interface design is the SA theory. Since the SA theory values both explanation and prediction, it has been widely applied in interface research to validate the effectiveness of UI design (e.g., [11,14,15]).

The concept of SA originated in research on fighter pilots in the 1990s (e.g., [16]) to explain the psychological processes of pilots in complex and dynamic environments. Then it was extended to other scenarios [17]. In such studies, the involvement of SA and the related approaches brought considerable benefits such as high efficiency and error reduction [18]. Therefore, exploring the characteristics of interfaces based on SA theory is a feasible approach.

There are three distinct definitions of SA theory: (i) individual SA, (ii) team SA, and (iii) system SA. Individual SA means “knowing what is going on around you” [18]. SA is not an entity that can be touched and observed, but rather a concept involving a cognitive black box. Therefore, there is no unified definition or measurement for it. One of the most famous individual SA models is the three-level model proposed by Endsley [19]. Endsley believed that SA was the individual’s perception of the elements of their environment, the comprehension of their meaning, and the projection of future states under specific time and space conditions. More specifically, the three levels were presented as follows:

Perception (Lv. 1): the simple awareness of task-related elements (objects, events, people, etc.) and their present states (locations, conditions, modes, actions, etc.) in the surrounding environment.

Comprehension (Lv. 2): Integrating elements from Lv. 1 through understanding their past states and how they impact goals or objectives.

Projection (Lv. 3): Integrating Lv. 1 and Lv. 2 information and using it to project future actions and states of the elements in the environment.

The Situation Awareness Global Assessment Technique (SAGAT), which was proposed based on the above definition, is a popular method of assessing individual SA through probe techniques [20]. Researchers need to compile situation-related questions on three levels: perception, comprehension, and projection. The test is inserted into the task. At this time, the task is suspended and the participants cannot view other information, which means they need to complete the test by memory. After the test is over, the task continues. Its reliability and validity have been confirmed in many experimental studies [14,21–23].

### The 3-player diner’s dilemma and interface design

**Fig 1:**
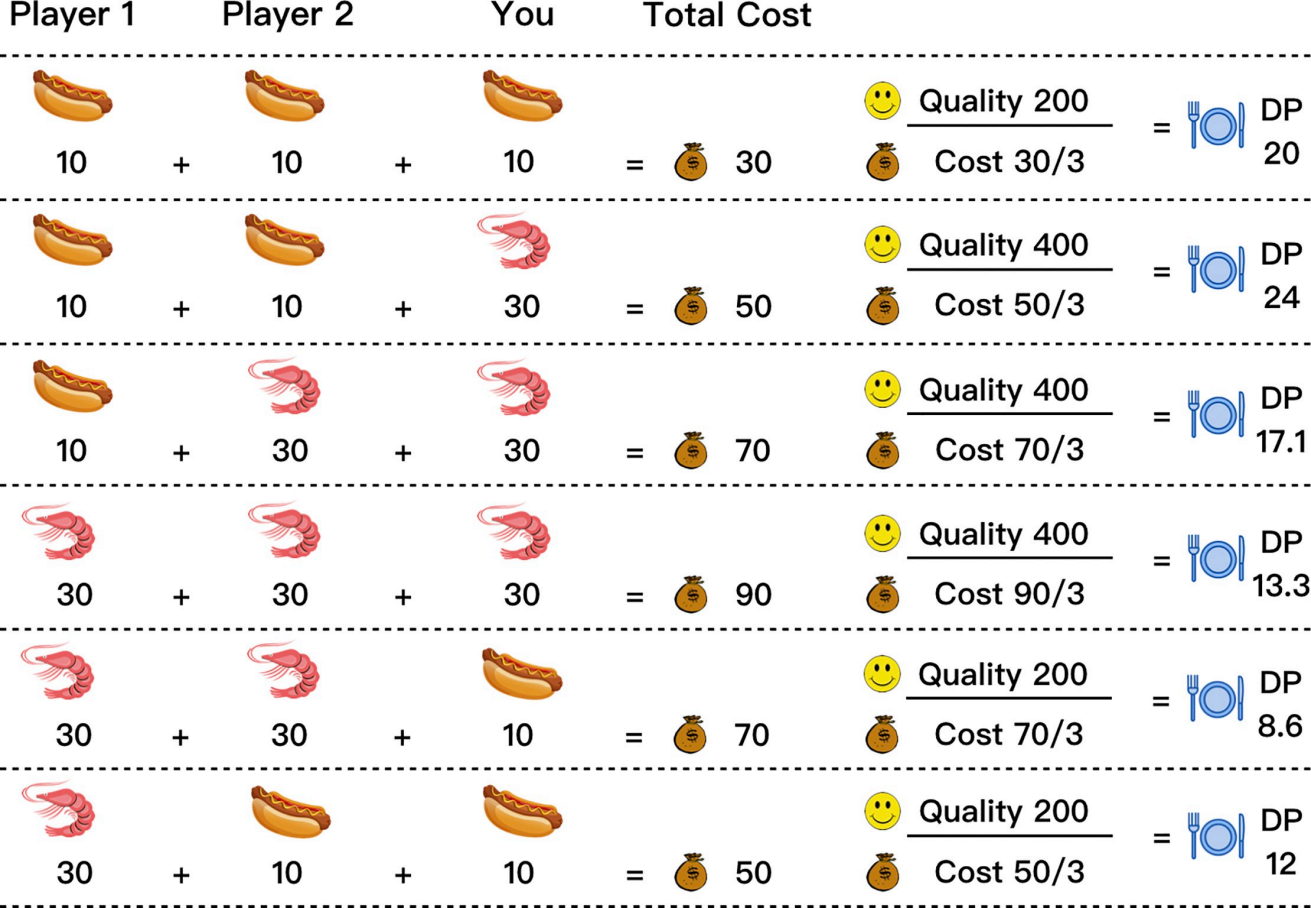
Dining points (DPs) and all possible outcomes in the game. In our study, there were six possible outcomes for each player.

In the 3-player diner’s dilemma, three players enter a restaurant. They agree to order a dish for themselves and the final cost is shared. The restaurant offers two types of dish, one is a hot dog at a low price and the other is a lobster at a high price. The more expensive the dish, the higher the quality. However, the hot dog has a higher quality-cost ratio. Here, we call this ratio dining points (DPs). In this game, players need to take part in multiple rounds of the game, with the goal of improving their total DPs. In our experiment, the hot dog had a quality of 200 and a cost of 10, so the DPs were 20, while the lobster had a quality of 400 and a cost of 30, so the DPs were 13.33. Since the final cost was shared by all three players, there were six possible outcomes for each player (see Fig 1).

Looking at Fig 1, we can see that although the hot dog had higher DPs, if the human player chose lobster and the other two computer players still chose the hot dog, the player could get more DPs from the loss of the other two players (see lines 1 and 2). However, if one of the computer players chose the lobster, the human player’s benefit would disappear or they would make a loss (see lines 3 and 4). In addition, if the other two computer players chose lobster and the human player still chose the hot dog, then their loss would be even greater (see lines 5 and 6).

Therefore, in order to get more DPs, the players need to observe how the other two behave. In the simplest case, if the other two players always chose the hot dog, the lobster would be the best choice (DPs = 24>20). Similarly, if the other two players always chose lobster, the lobster would also be the optimal solution (DPs = 13.3>8.6). So, when would the hot dog be the optimal solution? The answer is when the other two players were playing TFT (tit-for-tat). When the human player tried to get more DPs by selecting the lobster, the two computer players immediately chose the lobster to counterattack, so the human player’s total DPs would be reduced.

In summary, the players needed to understand the mathematical rules behind the situation and analyze the selection habits of the other players to develop their own strategies. The strategic depth of the game allowed us to design different interfaces to influence the task performance and SA of the participants. At the perception level (Lv. 1), the interface would present the basic task-related elements and allow the human player to understand their meaning. Specifically, all of the player's choices in the last round, as well as the player’s DPs, would be presented. All of this information was necessary for the player to complete the task. At the comprehension level (Lv. 2), as mentioned above, the tendencies of the other two players were vital to the task performance. Therefore, the information in UI-2 needed to reflect the tendencies of the other two players, especially whether they had been playing TFT. At the projection level (Lv. 3), the player needed to be able to use the information to predict future outcomes. The elements in UI-3 had to help the participants understand the possible outcomes of the different options. Because the three levels of SA should not be isolated, the UI-3 needed to contain all of the elements of the subordinate interfaces. So, we designed three interfaces for the game.

### Previous studies

As far as we know, a total of three studies used a similar experimental paradigm to explore the impact of UI on SA and task performance in the 3-player diner’s dilemma [23]. All of the previous studies were based on the three-level model of SA. Three levels of interface were designed. UI-1 involved the level of perception, UI-2 involved the level of comprehension, and UI-3 involved the level of projection. Participants played the game against two computer players.

Yun et al. [23] originally conducted the experiment to explore the relationship among

SA, trust, and interface types, laying the foundation for subsequent research. The results showed that when using UI-1, the participants had a higher tendency to cooperate, and in the context of encouraging cooperation, the self-reported trust scores and proportion of cooperation responses were positively correlated. However, as a first try, this study unavoidably had some limitations. First, SA was not measured directly. In the study, the interface types and levels of SA were made equivalent, but the interface types did not necessarily reflect the SA. Second, the computer strategy was not described in detail. Finally, the interface was relatively simple.

Therefore, in the study by Onal et al. [21], some important improvements were made. First, the researchers employed the SAGAT to obtain objective SA scores, making the SA and interface types no longer equivalent. Second, they tried to quantify the computer strategy so that the relationship between it and the behavior of the participants could be clearly recorded. Third, the interface design became more sophisticated. As a result, the study showed that the interface did significantly affect task performance and a significant positive correlation between SA and task performance was also detected.

In the latest research, Schafffer et al. [22] refined the study by upgrading the number of computer strategies (from 5 to 12), enlarging the sample population (from 95 to 901), and adding advanced statistical methods such as path analysis to explain the results. In general, the conclusions of the study were similar to those of Onal et al. [21]. The impact of the interface on SA was determined, and the SA scores increased along with the interface level. It was also found that the interface did affect task performance. In the context of encouraging cooperation, better performance tended to increase (indicating improved performance) with the increasing interface level.

In conclusion, past studies explained the relationship among interface, SA, and cooperation, but they still had some shortcomings. First, they did not introduce the time dimension, so it was impossible for them to explain whether (and how) task performance and SA would change with practice. That is to say, the above studies only considered the situation of "novices" in the diner’s dilemma. In fact, the “novice-expert” comparison was an important research issue for individual SA [17]. Also, Gonzalez et al. [14] have already shown that SA scores and task performance increased with practice in the water purification plant task. In their study, it was also found that only the levels of perception and comprehension increased with practice, while the third level of projection did not. Gonzalez et al. argued that this might be linked to the difficulty of the task. The establishment of a mental model had a positive effect on SA [24] and the essence of the practice effect might be the establishment of a mental model that could distinguish novices and experts [17]. So, it was necessary to probe the practice effect in different tasks to verify the assumption. Second, the computer strategies of the past studies still had a subdivision space. They set computer strategies through two types of parameter, distinguishing between cooperation and defection situations. These settings only changed one of the parameters while fixing the other, which was only a one-dimensional change. Therefore, our study extended it to a two-dimensional plane, making the computer strategy more similar to human behavior.

### Hypotheses

Based on the previous studies and literature, we proposed the following hypotheses:

H1: SA is positively correlated with task performance.
H2: As more components become involved in the interface, task performance will improve.

H3: The practice effect will exist in all three interface conditions.

## Method

The East China Normal University Ethics Committee approved this study. Our whole research process followed the ethical guidelines for research involving human subjects, and every participant provided written informed consent.

### Experimental design

This study adopted a mixed 3 (between-subjects variable) × 4 (within-subjects variable) design to examine the effects of interface and practice on DPs and SA scores. In the experiment, the participants were asked to compete with two computers that used a preset strategy, and they were encouraged to get as many DPs as possible. The task was divided into 4 blocks in a total of 200 trials. The program settled the DPs once every 50 trials, and then the DPs returned to zero to start a new block. The experiment contained three levels of UI, and each participant fulfilled the task under only one.

#### Computer strategy

In the experiment, the two computer players used the TFT strategy that was proposed by previous studies [21–23]. The TFT strategy meant that the computer would make the next round’s decisions based on the choice of the participant. In other words, if the participant chose a hot dog, the computer would also choose the hot dog in the next trial. Or if the participant chose the lobster, the computer would choose the lobster in the next trial. However, there was a 10% chance that the computer would make a different choice from the participant. There were two reasons for this setting: first, it ensured that the game had a clear optimal strategy so that the participants had the chance to learn; second, the probability was variable, which would make the computer players more natural, thus improving the ecological validity of the experiment.

#### Blocks and trials

**Fig 2:**
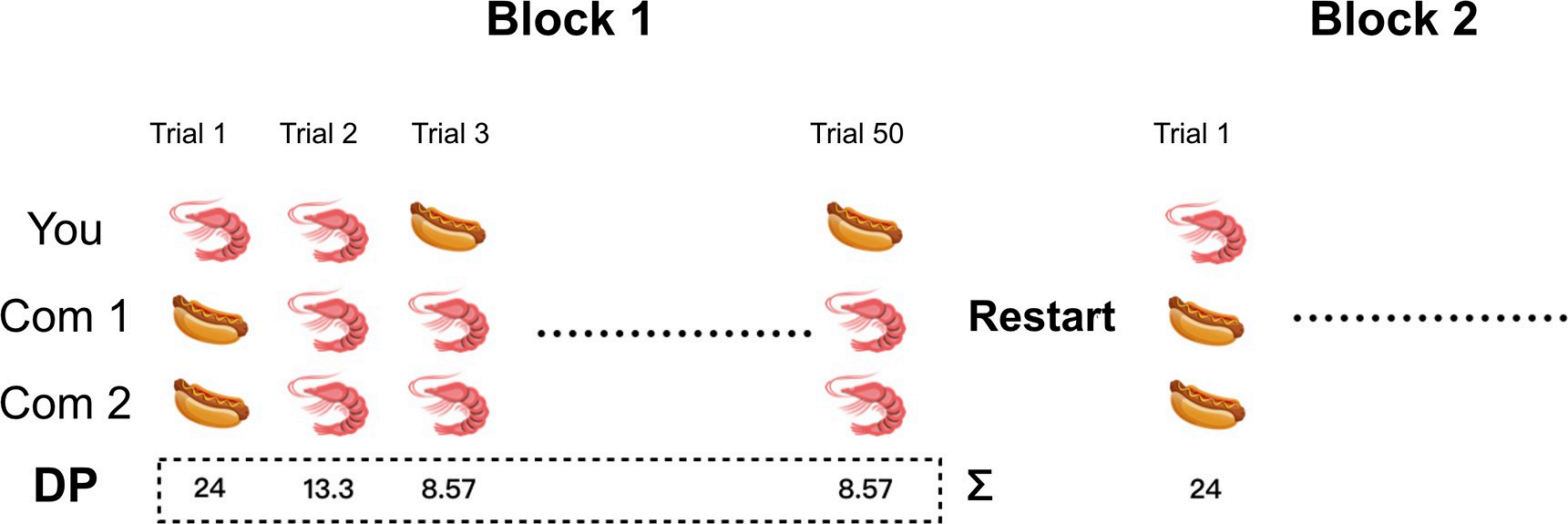
Blocks and trials settlement. In this study, the task was divided into 4 blocks, 50 trials per block. Dining points would be set to zero at the end of each block.

In order to verify the practice effect, the task was divided into 4 blocks, 50 trials per block, for a total of 200 trials. At the end of each block, the DP results (including all previous blocks) were presented to the participants and reset to 0 after confirmation (see Fig 2).

#### UI level

**Fig 3:**
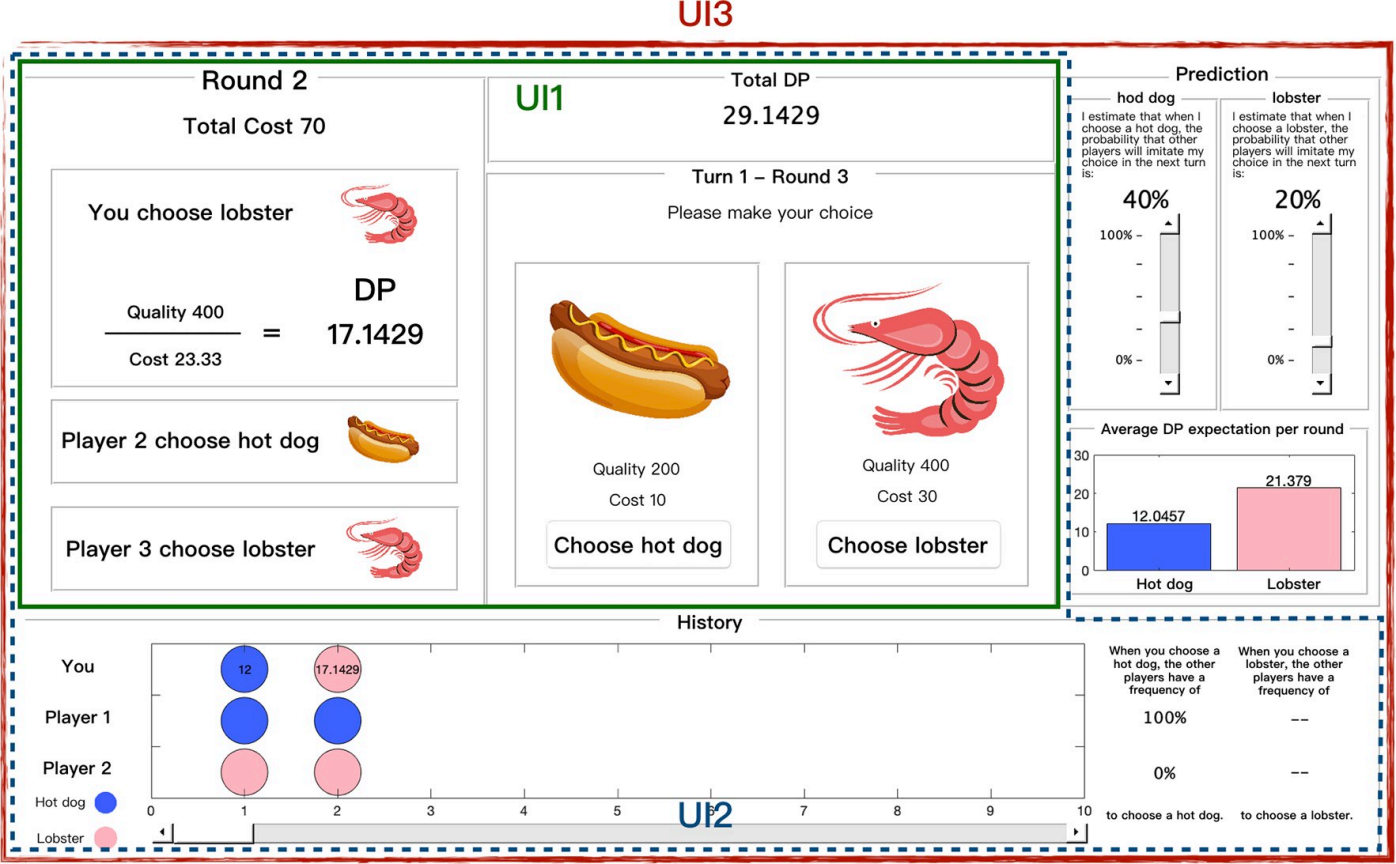
Integral user interface (UI). This figure shows the UIs employed in the study. The solid green line box is the level-1 UI, the blue dashed box is the level-2 UI, and the red line box is the level-3 UI.

The effect of the interface level was another focus of the experiment. Three levels were designed based on SA theory (see Fig 3).

Level-1 UI: The green solid line box shown in Fig 3 was a simplified UI (level-1 UI), in which only the basic buttons, the players’ selection for each trial, the DPs obtained in each trial, and the sum of the DPs were presented. At this level, participants could not directly view the selection tendency of the computer players.

Level-2 UI: The blue dashed box shown in Fig 3 was the level-2 UI. Compared with the level-1 UI, a history panel was added. In the history panel, the participants could view the selection information of all players in each trial, the DPs obtained in each trial, and the frequency count of the computer players mimicking the choices of the participants.

Level-3 UI: The red line frame part of Fig 3 was the level-3 UI. Compared with the level-2 UI, a prediction panel was added. Through the prediction panel, the participants could adjust the parameters and the program would calculate the expected DPs according to the preset formula to help the participants make decisions.

#### SAGAT measurement

The SAGAT was employed in this study to measure the SA scores. The test was inserted in the 25th trial of the task of every block. At this time, the participant could not view the main interface. According to the task situation, eight questions were put forward, of which questions 1–3 corresponded to the level of perception, 4–5 to the level of comprehension, and 6–8 to the level of projection. All were 4-to-1 single-choice questions, with 1 point for the correct choice and 0 points for the wrong choice, so the highest SA score for each test was 8 points. The eight questions are shown in Table 1.

**Table 1:** SAGAT questions.

No.	SA	Question
1	1	Up until now, what was the proportion of hot dogs you had chosen?
2	1	Up until now, what was the ratio of player 1 choosing hot dogs?
3	1	Up until now, what was the ratio of player 2 choosing hot dogs?
4	2	In which of the following situations was the sum of DPs each player received the largest?
5	2	In which of the following cases was the sum of DPs each player received the smallest?
6	3	If you had been choosing a hot dog since then, what was the average expected DP for each round?
7	3	If you had been choosing a lobster since then, what was the average expected DP for each round?
8	3	Which of the following strategies would allow you to get the most DPs?

### Equipment

The program was run on a MacBook Pro 13 with an external 25-inch 2560×1440 display. In the experiment, the participants used an external display and a Bluetooth mouse. Both the GUI and the background code were written in MATLAB 2019a.

### Participants

Initially, we recruited 90 undergraduates. Then, during the revision of the manuscript, we recruited another 27 undergraduates to improve the statistical power. Finally, a total of 117 undergraduates were recruited and formally informed, including 82 females and 35 males. The 117 volunteers (between 18 and 26 years old) were randomly assigned to 1 of the 3 interface conditions. Each group contained 39 participants. In the three interface conditions, the number of males was 11 (UI-1), 12 (UI-2), and 12 (UI-3).

## Results

### DPs

**Fig 4:**
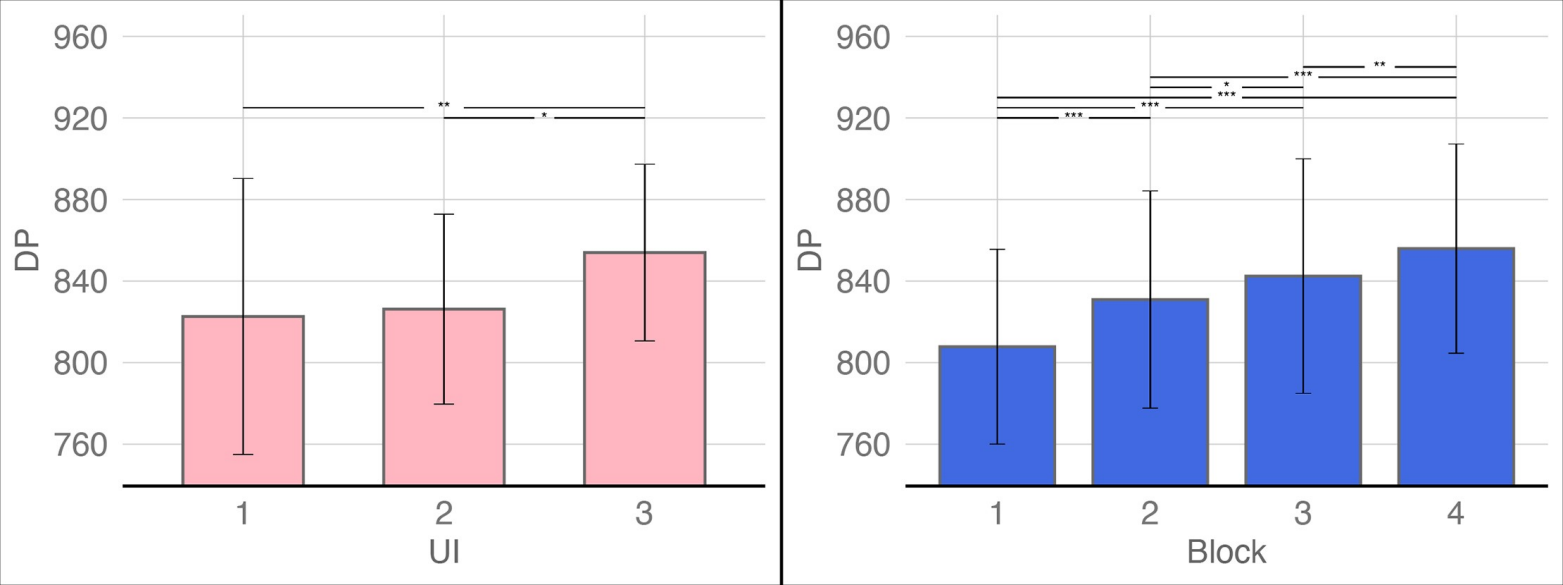
Bonferroni’s post-hoc comparisons of the main effects of block and UI level on DPs. The red box on the left of the figure shows the post-hoc comparison results of the block and the blue box on the right shows the post-hoc comparison results of the UI. (*p < 0.05, **p < 0.01, ***p < 0.001; the error bars denote 2 SDs).

A repeated measures analysis of variance (ANOVA) was employed to analyze the DPs. The results showed that the main effect of the block was significant: *F*(3, 342) = 48.924, *p* < 0.001, *η*_*partial*_^2^ = 0.300. The main effect of the interface condition was also significant: *F*(2, 114) = 6.183, *p* = 0.003, *η*_*partial*_^2^ = 0.098. However, the interaction between the block and the interface condition was not significant: *F*(6, 342) = 0.872, *p* = 0.515, *η*_*partial*_^2^ = 0.015. Post-hoc multiple comparisons with Bonferroni correction indicated that (1) there were significant differences between block 1 and block 2 (*p* < 0.001), block 1 and block 3 (*p* < 0.001), block 1 and block 4 (*p* < 0.001), block 2 and block 3 (*p* = 0.01), block 2 and block 4 (*p* < 0.001), and block 3 and block 4 (*p* = 0.002); (2) the DPs in UI-3 were significantly higher than in UI-1 (*p* = 0.005) and UI-2 (*p* = 0.016) and there were no significant differences between UI-1 and UI-2 (*p* = 1.000) (see Fig 4).

### SA scores

**Fig 5:**
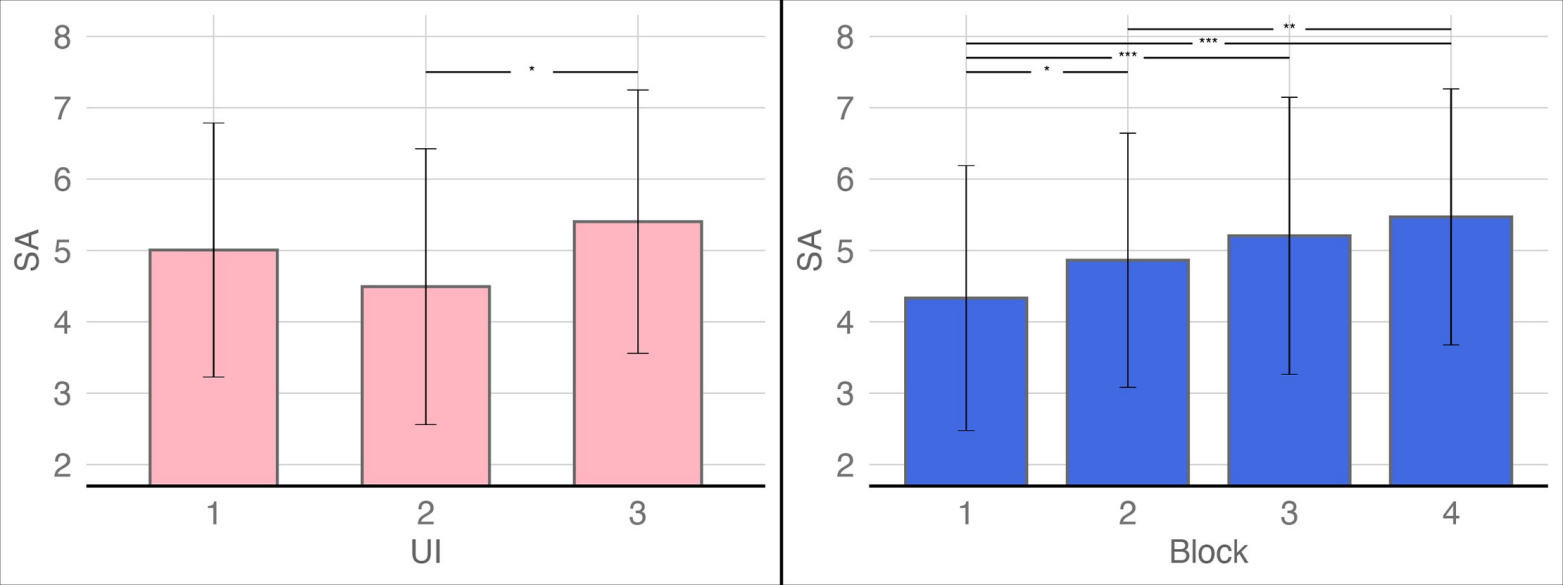
Bonferroni’s post-hoc comparisons of the main effects of block and UI level on situation awareness (SA) scores. The red box on the left of the figure shows the post-hoc comparison results of the block and the blue box on the right shows the post-hoc comparison results of the UI. (*p < 0.05, **p < 0.01, ***p < 0.001; the error bars denote 2 SDs).

Similarly, a repeated measures ANOVA was used to analyze the SA scores. The results showed that the main effect of the block was significant: *F*(3, 342) = 16.681, *p* < 0.001, *η*_*partial*_^2^ = 0.128. The main effect of the interface condition was also significant: *F*(2, 114) = 3.985, *p* = 0.021 < 0.05, *η*_*partial*_^2^ = 0.065. However, the interaction between the block and the interface condition was not significant: *F*(6, 342) = 0.631, *p* = 0.705, *η*_*partial*_^2^ = 0.011. Post-hoc multiple comparisons with Bonferroni correction indicated that (1) the SA scores of block 1 and block 2 (*p* = 0.027), block 1 and block 3 (*p* < 0.001), block 1 and block 4 (*p* < 0.001), and block 2 and block 4 (*p* = 0.001) showed significant differences and there were no significant differences between block 2 and block 3 (*p* = 0.228) or block 3 and block 4 (*p* = 0.289); (2) the SA scores of UI-3 were significantly higher than those of UI-2 (*p* = 0.017), while there were no significant differences between UI-1 and UI-3 (*p* = 0.664) and UI-1 and UI-2 (*p* = 0.346) (see Fig 5).

### Pearson’s correlation

**Fig 6:**
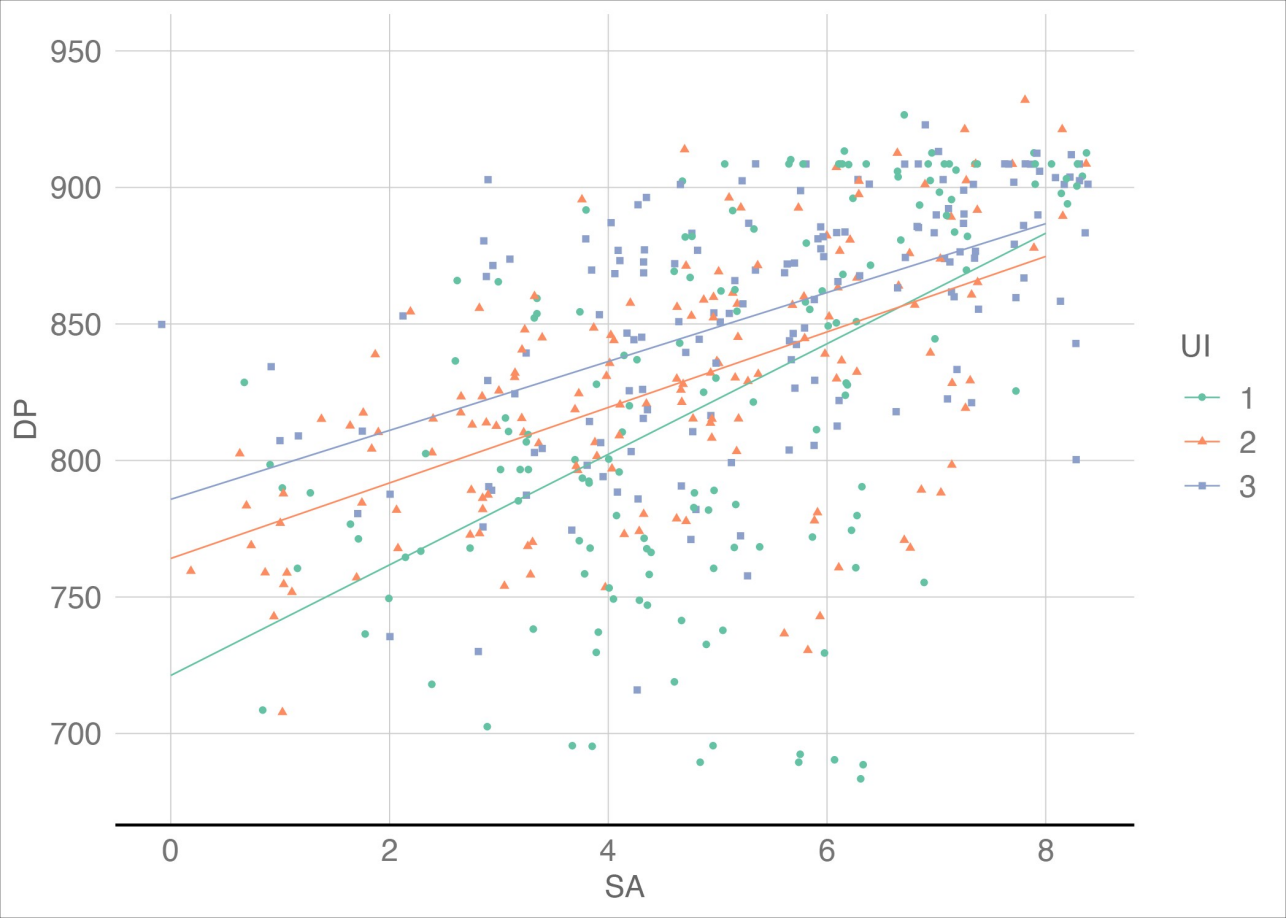
Correlation between SA scores and DPs. There was a significant positive correlation between SAs and DPs with *r* = 0.5, indicating that the SA scores could reflect the participants' understanding of the task.

In theory, both the SA scores and the DPs could reflect the participants' understanding of the task, and there should be a positive correlation between the two variables. The results showed that there did exist a significant positive correlation; the Pearson’s correlation coefficient results were *r* = 0.543, *p* < 0.001 (see Fig 6).

## Discussion

In our study, we examined the influence of interface design on task performance and SA, and also took the practice effect into consideration. There were two different dependent variables, in which the DPs reflected the task performance, while the SA scores reflected the understanding of the task. As mentioned above, the task in our study was not complicated in operation, so we had supposed that there would be a positive correlation between the two dependent variables (Hypothesis 1). The results supported the hypothesis, as there was a significant positive correlation (*r* = 0.543) between the DPs and the SA scores. High SA scores might have helped the participants emphasize the long-term gains over short-term gains (choosing lobster to gain more DPs in a single round), thus increasing the total DPs. On the other hand, the results also showed that the SAGAT had good reliability and validity, which could reflect the participants' understanding of the task.

The second hypothesis was that as more components became involved in the interface, the task performance would improve. In our interface design, UI-3 was the most complex level, which covered the compositions of the subordinate levels. Therefore, we speculated that UI-3 would lead to the best task performance and SA scores. The results verified this point. It was found that there was a significant positive effect of UI-3 on both the DPs and the SA scores. Therefore, the design of the prediction panel did enhance the participants' comprehension of the task situation. It not only helped the participants understand the rules more clearly, but also facilitated their more accurate and finer future planning. These results were in line with our hypothesis.

Taking a deeper view on this, the advantage of UI-3 might have come from the high integration of information. A good interface should not only integrate information but also embrace simplicity [25]. There was evidence that integrating all kinds of sub-UIs into a single UI was helpful for improving SA [26]. According to Durso et al., both working memory and mental models are relevant to SA [17]. By providing a graphical depiction of the diners’ historical decisions, UI-3 served as a working memory assist, which reduced the unnecessary cognitive load of the participants. Meanwhile, the prediction panel made good use of the information provided by the history panel, prompting the participants to build a correct mental model of the situation. The participants could understand the task through a single interface. So, under this condition, the participants performed better in both DPs and the SA test.

However, when using UI-2, there was no significant improvement in task performance and SA scores compared with UI-1. So, contrary to the effect of the prediction panel of UI-3, the history panel in UI-2, which corresponded to the comprehension level of SA theory, was not ideal. The history panel failed to improve the participants’ DPs or SA scores. Also, in the SAGAT, the SA scores of UI-2 were slightly lower than those of UI-1. The history panel also failed to help the participants answer the two questions that were relevant to the comprehension level. This result did not fit our hypothesis, but might reflect that more information was not always beneficial [7,11]. Looking back at UI-2, the frequency count on the history panel did not have an intuitive effect in the absence of a predictive panel, which might have made the participants feel puzzled. This also suggested that simply providing additional information did not necessarily improve SA and task performance.

The third hypothesis was that the practice effect would exist in all three interface conditions. The results showed that it did exist in all three conditions; not only the DPs, but also the SA scores significantly increased with practice. Also, the levels of UI had no effect on the task learning process, which implied that the learning rules remained consistent across the different conditions. According to Endsley, it usually takes a long time to build SA [27]. However, in a simple task, the SA could also change through training in a short time [28]. The task of this experiment should belong to the latter, as the task was a relatively simple simulation scenario, so the SA scores could change significantly in the four blocks. However, the improvement of the DPs and the SA scores was not smooth. The SA scores tended to be stable in blocks 2, 3, and 4, while the changes in DPs were significant from block to block. These results implied that the participants had established a stable mental model of the task. In addition, no interaction of blocks and UI level was found in the experiment. Given that the assignment of the participants was completely random, it could be inferred that the pre-task instructions and practice were the main reasons why the UI-3 group had the best performance. In the subsequent whole process of the task, the advantages were continually maintained, neither expanding nor decreasing. This might mean that the UI level did not have a substantial impact on the efficiency of task learning, and the design of the UI might not have changed the law of learning.

### Limitations

First, the main purpose of our study was to verify the theoretical basis for interface design. So, we adopted the 3-player diner's dilemma as the experimental task, which was a simulated situation. Moreover, we chose only situations where the computer played TFT without considering more complex computer strategies, such as computer players dynamically adjusting their preferences in response to the participants’ choices. Given that, whether our results could be extended to other tasks or even to the real world still remains to be proven.

Another limitation is that our interface design did not exclude all of the irrelevant variables. UI-3 was interactive, while UI-1 and UI-2 were not. Previous studies confirmed that the controllability of the system was beneficial to understanding the situation and improving task performance and satisfaction [13,29]. Thus, it is the interaction itself that might help the participants to have better control over the UI and deepen their understanding of the task. To eliminate this possibility, in the future, we would like to change the parameter adjustment in the prediction panel in UI-3 from manual input to automatic input to test this assumption.

Finally, the SAGAT in our study used only eight questions, which could have led to insufficient reliability and validity of the test. Specifically, even if the participants did not know the correct answer, just guessing might have led to a high score. In addition, the effect size of the SA became small. Therefore, in future experiments, we might add more questions and refine the test to improve its reliability.

## Conclusions

Three interfaces based on the three-level SA model were designed to explore the role of interface design in task performance, SA scores, and the learning process in a simulated situation. We found that: (1) the level-3 interface effectively improved the task performance and SA scores, while the level-2 interface failed to improve them; (2) the practice effect did exist in all three conditions; and (3) the levels of UI had no effect on the task learning process, which implied that the learning rules remained consistent across the different conditions.

## Supporting information

S1 Table Descriptive statistical results of the main variables of the experiment. (DOCX)

S1 Appendix Mixed linear model [30–31]. (DOCX)

S1 Raw dataset (DOCX)
