# Supplementary material for: The influence of user interface design on task performance and situation awareness in a 3-player diner's dilemma game
Source: PLoS One. 2020 Mar 17;15(3):e0230387. doi: 10.1371/journal.pone.0230387 (PMC7077814; doi:10.1371/journal.pone.0230387)
Supplement: S1 Table — (DOCX) [file pone.0230387.s001.docx]

**S1 Table**. **Descriptive statistical results of the main variables of the experiment**

| Block | UI | DP | SA score |
| --- | --- | --- | --- |
| 1 | 1 | 792.86±58.47 | 4.49±1.76 |
| 1 | 2 | 804.11±37.59 | 3.87±1.88 |
| 1 | 3 | 826.50±39.22 | 4.64±1.89 |
| 2 | 1 | 815.37±64.54 | 4.69±1.32 |
| 2 | 2 | 821.45±43.22 | 4.36±1.99 |
| 2 | 3 | 855.99±40.57 | 5.54±1.79 |
| 3 | 1 | 832.09±70.55 | 5.23±1.98 |
| 3 | 2 | 834.62±48.28 | 4.72±1.97 |
| 3 | 3 | 860.60±47.63 | 5.67±1.80 |
| 4 | 1 | 850.21±65.49 | 5.62±1.83 |
| 4 | 2 | 844.81±47.70 | 5.03±1.75 |
| 4 | 3 | 872.82±31.94 | 5.77±1.75 |
