# Supplementary material for: The influence of user interface design on task performance and situation awareness in a 3-player diner's dilemma game
Source: PLoS One. 2020 Mar 17;15(3):e0230387. doi: 10.1371/journal.pone.0230387 (PMC7077814; doi:10.1371/journal.pone.0230387)
Supplement: S1 Appendix — (DOCX) [file pone.0230387.s002.docx]

## S1 Appendix. Mixed Linear Model

Besides a repeated measure ANOVA, a mixed linear model could also be employed to analyze the data. A mixed linear model is an extension of a linear model, which is becoming immensely popular as a framework for the analysis of longitudinal data [30]. We used the baseline of each participant as the intercept in the regression model. According to our assumption, we got the following models:

$$\begin{aligned} DP \sim Block*UI+(1│Subject)+\varepsilon\#\left( 3 \right) \end{aligned}$$

$$\begin{aligned} SA \sim Block*UI+(1│Subject)+\varepsilon\#\left( 4 \right) \end{aligned}$$

Here, (1|Subject) is the different intercept generated for each participant's baseline performance. The block is treated as a continuous predictor to increase statistical power. We used the lnm4 and lmerTest package in R to analyze the data. The results are shown in Tables 1 and 2.

**S2 Table 1**. **The results on dining points (DPs) by the mixed liner model**

| Random effects | Variance | Std. |  |  |  |
| --- | --- | --- | --- | --- | --- |
| Subjects | 1561.2 | 39.51 |  |  |  |
| Residual | 985.1 | 31.39 |  |  |  |
| Fixed effects | Estimate | Error | df | t | p |
| (Intercept) | 775.44 | 8.83 | 286.41 | 87.847 | <0.001*** |
| UI1 (baseline) |  |  |  |  |  |
| UI2 | 16.99 | 12.48 | 286.41 | 1.361 | 0.175 |
| UI3 | 42.65 | 12.48 | 286.41 | 3.416 | <0.001*** |
| Block | 18.88 | 2.25 | 351.00 | 8.398 | <0.001*** |
| UI2*Block | -5.35 | 3.18 | 351.00 | -1.682 | 0.093 |
| UI3*Block | -4.52 | 3.18 | 351.00 | -1.422 | 0.156 |

**S2 Table 2**. **The results on situation awareness (SA) scores by the mixed liner model**

| Random effects | Variance | Std. |  |  |  |
| --- | --- | --- | --- | --- | --- |
| Subjects | 1.568 | 1.252 |  |  |  |
| Residual | 1.669 | 1.292 |  |  |  |
| Fixed effects | Estimate | Error | df | t | p |
| (Intercept) | 4.03 | 0.32 | 359.63 | 12.459 | <0.001*** |
| UI1 (baseline) |  |  |  |  |  |
| UI2 | -0.49 | 0.46 | 359.63 | -1.066 | 0.287 |
| UI3 | 0.50 | 0.46 | 359.63 | 1.094 | 0.275 |
| Block | 0.39 | 0.09 | 351.00 | 4.241 | <0.001*** |
| UI2*Block | -0.01 | 0.13 | 351.00 | -0.078 | 0.938 |
| UI3*Block | -0.04 | 0.13 | 351.00 | -0.314 | 0.754 |

Note that since we set UI1 as the baseline in the regression, it was unclear whether performance under the UI3 differed from that of UI2. To make the results of the mixed linear model comparable with the results of the repeated measures ANOVA, we set UI2 as the baseline for regression, and the results are shown in the Tables 3 and 4.

**S2 Table 3**. **The results on DPs by the mixed liner model (baseline = UI2)**

| Random effects | Variance | Std. |  |  |  |
| --- | --- | --- | --- | --- | --- |
| Subjects | 1561.2 | 39.51 |  |  |  |
| Residual | 985.1 | 31.39 |  |  |  |
| Fixed effects | Estimate | Error | df | t | p |
| (Intercept) | 792.43 | 8.83 | 286.41 | 89.771 | <0.001*** |
| UI1 | -16.99 | 12.48 | 286.41 | -1.361 | 0.175 |
| UI2 (baseline) |  |  |  |  |  |
| UI3 | 25.66 | 12.48 | 286.41 | 2.056 | 0.041* |
| Block | 13.53 | 2.25 | 351.00 | 6.019 | <0.001*** |
| UI1*Block | 5.35 | 3.18 | 351.00 | 1.682 | 0.093 |
| UI3*Block | 0.83 | 3.18 | 351.00 | 0.260 | 0.795 |

**S2 Table 4**. **The results on SA scores by the mixed liner model (baseline = UI2).**

| Random effects | Variance | Std. |  |  |  |
| --- | --- | --- | --- | --- | --- |
| Subjects | 1.568 | 1.252 |  |  |  |
| Residual | 1.669 | 1.292 |  |  |  |
| Fixed effects | Estimate | Error | df | t | p |
| (Intercept) | 3.54 | 0.32 | 359.63 | 10.951 | <0.001*** |
| UI1 | 0.49 | 0.46 | 359.63 | 1.066 | 0.287 |
| UI2 (baseline) |  |  |  |  |  |
| UI3 | 0.99 | 0.46 | 359.63 | 2.160 | 0.031* |
| Block | 0.38 | 0.09 | 351.00 | 4.130 | <0.001*** |
| UI1*Block | 0.01 | 0.13 | 351.00 | 0.078 | 0.938 |
| UI3*Block | -0.03 | 0.13 | 351.00 | -0.235 | 0.814 |

The results were consistent with those of the repeated measure ANOVA. Given that the significance test in mixed linear modelling is controversial [31], we still used a traditional repeated measures ANOVA in the main body. Therefore, the methods adopted here should be for reference only.
