# Supplementary material for: The influence of user interface design on task performance and situation awareness in a 3-player diner's dilemma game
Source: PLoS One. 2020 Mar 17;15(3):e0230387. doi: 10.1371/journal.pone.0230387 (PMC7077814; doi:10.1371/journal.pone.0230387)
Supplement: S1 Raw dataset — (DOCX) [file pone.0230387.s003.docx]

## Raw dataset

| Subject | Block | UI | DP | SA |
| --- | --- | --- | --- | --- |
| 1 | 1 | 1 | 760.76 | 6 |
| 1 | 2 | 1 | 767.62 | 4 |
| 1 | 3 | 1 | 770.48 | 4 |
| 1 | 4 | 1 | 774.48 | 6 |
| 2 | 1 | 2 | 770.86 | 7 |
| 2 | 2 | 2 | 825.52 | 3 |
| 2 | 3 | 2 | 907.43 | 6 |
| 2 | 4 | 2 | 896.19 | 5 |
| 3 | 1 | 3 | 883.43 | 6 |
| 3 | 2 | 3 | 874.1 | 7 |
| 3 | 3 | 3 | 885.71 | 7 |
| 3 | 4 | 3 | 839.62 | 5 |
| 4 | 1 | 1 | 738.29 | 3 |
| 4 | 2 | 1 | 771.43 | 4 |
| 4 | 3 | 1 | 796.57 | 3 |
| 4 | 4 | 1 | 823.81 | 6 |
| 5 | 1 | 2 | 832 | 5 |
| 5 | 2 | 2 | 889.52 | 8 |
| 5 | 3 | 2 | 921.33 | 8 |
| 5 | 4 | 2 | 932 | 8 |
| 6 | 1 | 3 | 815.43 | 4 |
| 6 | 2 | 3 | 877.14 | 4 |
| 6 | 3 | 3 | 880.38 | 3 |
| 6 | 4 | 3 | 902.86 | 3 |
| 7 | 1 | 1 | 824.95 | 5 |
| 7 | 2 | 1 | 908.38 | 6 |
| 7 | 3 | 1 | 898.29 | 7 |
| 7 | 4 | 1 | 926.48 | 7 |
| 8 | 1 | 2 | 806.67 | 4 |
| 8 | 2 | 2 | 902.48 | 7 |
| 8 | 3 | 2 | 913.9 | 5 |
| 8 | 4 | 2 | 921.33 | 7 |
| 9 | 1 | 3 | 822.48 | 7 |
| 9 | 2 | 3 | 883.43 | 7 |
| 9 | 3 | 3 | 877.52 | 6 |
| 9 | 4 | 3 | 922.86 | 7 |
| 10 | 1 | 1 | 791.81 | 4 |
| 10 | 2 | 1 | 854.67 | 5 |
| 10 | 3 | 1 | 908.57 | 5 |
| 10 | 4 | 1 | 908.57 | 6 |
| 11 | 1 | 2 | 758.86 | 1 |
| 11 | 2 | 2 | 796.38 | 4 |
| 11 | 3 | 2 | 810.29 | 2 |
| 11 | 4 | 2 | 803.43 | 5 |
| 12 | 1 | 3 | 780.57 | 2 |
| 12 | 2 | 3 | 850.67 | 5 |
| 12 | 3 | 3 | 789.14 | 3 |
| 12 | 4 | 3 | 842.48 | 6 |
| 13 | 1 | 1 | 783.81 | 5 |
| 13 | 2 | 1 | 800.38 | 4 |
| 13 | 3 | 1 | 858.1 | 6 |
| 13 | 4 | 1 | 910.29 | 6 |
| 14 | 1 | 2 | 789.33 | 7 |
| 14 | 2 | 2 | 772.95 | 4 |
| 14 | 3 | 2 | 768 | 7 |
| 14 | 4 | 2 | 845.14 | 5 |
| 15 | 1 | 3 | 810.67 | 2 |
| 15 | 2 | 3 | 849.9 | 0 |
| 15 | 3 | 3 | 829.33 | 3 |
| 15 | 4 | 3 | 873.71 | 3 |
| 16 | 1 | 1 | 904 | 8 |
| 16 | 2 | 1 | 891.62 | 5 |
| 16 | 3 | 1 | 908.57 | 7 |
| 16 | 4 | 1 | 902.48 | 7 |
| 17 | 1 | 2 | 810.29 | 3 |
| 17 | 2 | 2 | 788.19 | 7 |
| 17 | 3 | 2 | 877.9 | 8 |
| 17 | 4 | 2 | 908.57 | 8 |
| 18 | 1 | 3 | 716 | 4 |
| 18 | 2 | 3 | 735.43 | 2 |
| 18 | 3 | 3 | 730.1 | 3 |
| 18 | 4 | 3 | 868.76 | 4 |
| 19 | 1 | 1 | 853.71 | 3 |
| 19 | 2 | 1 | 862.1 | 5 |
| 19 | 3 | 1 | 883.62 | 7 |
| 19 | 4 | 1 | 891.81 | 4 |
| 20 | 1 | 2 | 786.29 | 3 |
| 20 | 2 | 2 | 784.57 | 2 |
| 20 | 3 | 2 | 751.81 | 1 |
| 20 | 4 | 2 | 757.14 | 2 |
| 21 | 1 | 3 | 798.29 | 4 |
| 21 | 2 | 3 | 821.9 | 6 |
| 21 | 3 | 3 | 785.9 | 4 |
| 21 | 4 | 3 | 774.48 | 4 |
| 22 | 1 | 1 | 741.33 | 5 |
| 22 | 2 | 1 | 854.48 | 4 |
| 22 | 3 | 1 | 893.52 | 7 |
| 22 | 4 | 1 | 905.9 | 7 |
| 23 | 1 | 2 | 758.86 | 1 |
| 23 | 2 | 2 | 876.76 | 6 |
| 23 | 3 | 2 | 857.33 | 5 |
| 23 | 4 | 2 | 863.24 | 6 |
| 24 | 1 | 3 | 824.38 | 3 |
| 24 | 2 | 3 | 908.57 | 8 |
| 24 | 3 | 3 | 908.57 | 8 |
| 24 | 4 | 3 | 881.14 | 6 |
| 25 | 1 | 1 | 732.76 | 5 |
| 25 | 2 | 1 | 790.29 | 6 |
| 25 | 3 | 1 | 755.24 | 7 |
| 25 | 4 | 1 | 737.14 | 4 |
| 26 | 1 | 2 | 829.9 | 6 |
| 26 | 2 | 2 | 855.81 | 3 |
| 26 | 3 | 2 | 844.76 | 6 |
| 26 | 4 | 2 | 908.57 | 7 |
| 27 | 1 | 3 | 883.62 | 6 |
| 27 | 2 | 3 | 902.48 | 5 |
| 27 | 3 | 3 | 901.14 | 7 |
| 27 | 4 | 3 | 901.14 | 8 |
| 28 | 1 | 1 | 683.43 | 6 |
| 28 | 2 | 1 | 690.29 | 6 |
| 28 | 3 | 1 | 688.57 | 6 |
| 28 | 4 | 1 | 689.52 | 6 |
| 29 | 1 | 2 | 856.19 | 5 |
| 29 | 2 | 2 | 895.62 | 4 |
| 29 | 3 | 2 | 871.24 | 5 |
| 29 | 4 | 2 | 848.57 | 4 |
| 30 | 1 | 3 | 812.57 | 6 |
| 30 | 2 | 3 | 869.71 | 5 |
| 30 | 3 | 3 | 872 | 6 |
| 30 | 4 | 3 | 829.33 | 6 |
| 31 | 1 | 1 | 771.24 | 2 |
| 31 | 2 | 1 | 796.57 | 3 |
| 31 | 3 | 1 | 908.57 | 6 |
| 31 | 4 | 1 | 850.48 | 6 |
| 32 | 1 | 2 | 875.81 | 7 |
| 32 | 2 | 2 | 861.33 | 5 |
| 32 | 3 | 2 | 865.33 | 7 |
| 32 | 4 | 2 | 889.14 | 7 |
| 33 | 1 | 3 | 782.1 | 5 |
| 33 | 2 | 3 | 771.05 | 5 |
| 33 | 3 | 3 | 757.71 | 5 |
| 33 | 4 | 3 | 821.14 | 7 |
| 34 | 1 | 1 | 729.71 | 4 |
| 34 | 2 | 1 | 771.81 | 6 |
| 34 | 3 | 1 | 779.81 | 6 |
| 34 | 4 | 1 | 895.62 | 7 |
| 35 | 1 | 2 | 820.76 | 4 |
| 35 | 2 | 2 | 892.57 | 5 |
| 35 | 3 | 2 | 864 | 7 |
| 35 | 4 | 2 | 891.81 | 7 |
| 36 | 1 | 3 | 859.62 | 8 |
| 36 | 2 | 3 | 890.29 | 7 |
| 36 | 3 | 3 | 903.81 | 8 |
| 36 | 4 | 3 | 902.48 | 8 |
| 37 | 1 | 1 | 708.57 | 1 |
| 37 | 2 | 1 | 702.48 | 3 |
| 37 | 3 | 1 | 695.43 | 4 |
| 37 | 4 | 1 | 865.33 | 3 |
| 38 | 1 | 2 | 777.71 | 5 |
| 38 | 2 | 2 | 823.43 | 3 |
| 38 | 3 | 2 | 852.76 | 6 |
| 38 | 4 | 2 | 753.52 | 4 |
| 39 | 1 | 3 | 807.24 | 1 |
| 39 | 2 | 3 | 865.9 | 5 |
| 39 | 3 | 3 | 892.19 | 7 |
| 39 | 4 | 3 | 901.14 | 6 |
| 40 | 1 | 1 | 827.81 | 4 |
| 40 | 2 | 1 | 867.05 | 5 |
| 40 | 3 | 1 | 869.14 | 5 |
| 40 | 4 | 1 | 821.33 | 5 |
| 41 | 1 | 2 | 742.86 | 1 |
| 41 | 2 | 2 | 777.14 | 1 |
| 41 | 3 | 2 | 759.43 | 0 |
| 41 | 4 | 2 | 815.24 | 1 |
| 42 | 1 | 3 | 844.19 | 4 |
| 42 | 2 | 3 | 873.14 | 4 |
| 42 | 3 | 3 | 868.38 | 4 |
| 42 | 4 | 3 | 893.71 | 4 |
| 43 | 1 | 1 | 729.33 | 6 |
| 43 | 2 | 1 | 695.43 | 5 |
| 43 | 3 | 1 | 692.38 | 6 |
| 43 | 4 | 1 | 689.52 | 5 |
| 44 | 1 | 2 | 813.9 | 3 |
| 44 | 2 | 2 | 845.9 | 4 |
| 44 | 3 | 2 | 839.05 | 6 |
| 44 | 4 | 2 | 836.38 | 5 |
| 45 | 1 | 3 | 787.24 | 3 |
| 45 | 2 | 3 | 842.86 | 8 |
| 45 | 3 | 3 | 858.29 | 8 |
| 45 | 4 | 3 | 883.24 | 5 |
| 46 | 1 | 1 | 737.71 | 5 |
| 46 | 2 | 1 | 753.33 | 4 |
| 46 | 3 | 1 | 902.29 | 5 |
| 46 | 4 | 1 | 850.86 | 6 |
| 47 | 1 | 2 | 758.1 | 3 |
| 47 | 2 | 2 | 780.38 | 4 |
| 47 | 3 | 2 | 770.1 | 3 |
| 47 | 4 | 2 | 871.43 | 5 |
| 48 | 1 | 3 | 805.52 | 6 |
| 48 | 2 | 3 | 896.38 | 4 |
| 48 | 3 | 3 | 901.9 | 8 |
| 48 | 4 | 3 | 908.57 | 8 |
| 49 | 1 | 1 | 868.19 | 6 |
| 49 | 2 | 1 | 764.38 | 2 |
| 49 | 3 | 1 | 789.9 | 1 |
| 49 | 4 | 1 | 865.9 | 3 |
| 50 | 1 | 2 | 767.81 | 2 |
| 50 | 2 | 2 | 806.29 | 3 |
| 50 | 3 | 2 | 824.57 | 4 |
| 50 | 4 | 2 | 820.38 | 4 |
| 51 | 1 | 3 | 845.14 | 4 |
| 51 | 2 | 3 | 854.1 | 5 |
| 51 | 3 | 3 | 872.38 | 6 |
| 51 | 4 | 3 | 852.95 | 2 |
| 52 | 1 | 1 | 896 | 6 |
| 52 | 2 | 1 | 908.57 | 6 |
| 52 | 3 | 1 | 908.57 | 8 |
| 52 | 4 | 1 | 908.57 | 7 |
| 53 | 1 | 2 | 830.86 | 4 |
| 53 | 2 | 2 | 801.52 | 4 |
| 53 | 3 | 2 | 832.38 | 6 |
| 53 | 4 | 2 | 777.9 | 6 |
| 54 | 1 | 3 | 803.24 | 4 |
| 54 | 2 | 3 | 826.48 | 6 |
| 54 | 3 | 3 | 808.95 | 1 |
| 54 | 4 | 3 | 839.43 | 3 |
| 55 | 1 | 1 | 760.38 | 1 |
| 55 | 2 | 1 | 768 | 3 |
| 55 | 3 | 1 | 810.48 | 3 |
| 55 | 4 | 1 | 796.76 | 3 |
| 56 | 1 | 2 | 778.67 | 5 |
| 56 | 2 | 2 | 760.76 | 6 |
| 56 | 3 | 2 | 742.86 | 6 |
| 56 | 4 | 2 | 825.9 | 5 |
| 57 | 1 | 3 | 787.62 | 2 |
| 57 | 2 | 3 | 846.67 | 4 |
| 57 | 3 | 3 | 874.67 | 6 |
| 57 | 4 | 3 | 843.81 | 6 |
| 58 | 1 | 1 | 781.71 | 5 |
| 58 | 2 | 1 | 855.43 | 6 |
| 58 | 3 | 1 | 912.57 | 7 |
| 58 | 4 | 1 | 912.57 | 8 |
| 59 | 1 | 2 | 787.81 | 1 |
| 59 | 2 | 2 | 812.57 | 3 |
| 59 | 3 | 2 | 818.67 | 4 |
| 59 | 4 | 2 | 830.29 | 5 |
| 60 | 1 | 3 | 814.29 | 4 |
| 60 | 2 | 3 | 775.62 | 3 |
| 60 | 3 | 3 | 772.38 | 5 |
| 60 | 4 | 3 | 836.95 | 6 |
| 61 | 1 | 1 | 788.95 | 5 |
| 61 | 2 | 1 | 882.1 | 7 |
| 61 | 3 | 1 | 908.57 | 7 |
| 61 | 4 | 1 | 897.71 | 8 |
| 62 | 1 | 2 | 772.76 | 3 |
| 62 | 2 | 2 | 782.1 | 3 |
| 62 | 3 | 2 | 773.33 | 3 |
| 62 | 4 | 2 | 768.57 | 3 |
| 63 | 1 | 3 | 804.38 | 3 |
| 63 | 2 | 3 | 885.52 | 6 |
| 63 | 3 | 3 | 874.29 | 7 |
| 63 | 4 | 3 | 886.86 | 5 |
| 64 | 1 | 1 | 838.48 | 4 |
| 64 | 2 | 1 | 810.29 | 4 |
| 64 | 3 | 1 | 766.67 | 2 |
| 64 | 4 | 1 | 798.48 | 1 |
| 65 | 1 | 2 | 815.43 | 3 |
| 65 | 2 | 2 | 836.57 | 6 |
| 65 | 3 | 2 | 813.71 | 5 |
| 65 | 4 | 2 | 844 | 4 |
| 66 | 1 | 3 | 790.48 | 3 |
| 66 | 2 | 3 | 825.52 | 4 |
| 66 | 3 | 3 | 816.38 | 5 |
| 66 | 4 | 3 | 846.48 | 6 |
| 67 | 1 | 1 | 844.57 | 7 |
| 67 | 2 | 1 | 881.9 | 5 |
| 67 | 3 | 1 | 901.14 | 8 |
| 67 | 4 | 1 | 912.57 | 8 |
| 68 | 1 | 2 | 809.14 | 4 |
| 68 | 2 | 2 | 783.43 | 1 |
| 68 | 3 | 2 | 830.48 | 3 |
| 68 | 4 | 2 | 869.14 | 5 |
| 69 | 1 | 3 | 810.48 | 5 |
| 69 | 2 | 3 | 902.86 | 6 |
| 69 | 3 | 3 | 908.57 | 5 |
| 69 | 4 | 3 | 887.05 | 4 |
| 70 | 1 | 1 | 800.57 | 4 |
| 70 | 2 | 1 | 806.67 | 3 |
| 70 | 3 | 1 | 836.38 | 3 |
| 70 | 4 | 1 | 830.1 | 5 |
| 71 | 1 | 2 | 798.29 | 7 |
| 71 | 2 | 2 | 828.38 | 7 |
| 71 | 3 | 2 | 852.95 | 5 |
| 71 | 4 | 2 | 866.86 | 6 |
| 72 | 1 | 3 | 818.67 | 4 |
| 72 | 2 | 3 | 858.86 | 6 |
| 72 | 3 | 3 | 848.57 | 6 |
| 72 | 4 | 3 | 912 | 8 |
| 73 | 1 | 1 | 776.57 | 2 |
| 73 | 2 | 1 | 785.14 | 3 |
| 73 | 3 | 1 | 760.57 | 5 |
| 73 | 4 | 1 | 768 | 4 |
| 74 | 1 | 2 | 823.43 | 3 |
| 74 | 2 | 2 | 860 | 6 |
| 74 | 3 | 2 | 897.52 | 6 |
| 74 | 4 | 2 | 908.57 | 8 |
| 75 | 1 | 3 | 889.9 | 7 |
| 75 | 2 | 3 | 861.71 | 7 |
| 75 | 3 | 3 | 902.86 | 7 |
| 75 | 4 | 3 | 908.57 | 6 |
| 76 | 1 | 1 | 718.86 | 5 |
| 76 | 2 | 1 | 758.29 | 4 |
| 76 | 3 | 1 | 779.81 | 4 |
| 76 | 4 | 1 | 793.52 | 4 |
| 77 | 1 | 2 | 817.52 | 3 |
| 77 | 2 | 2 | 835.62 | 5 |
| 77 | 3 | 2 | 857.71 | 4 |
| 77 | 4 | 2 | 835.62 | 4 |
| 78 | 1 | 3 | 872 | 5 |
| 78 | 2 | 3 | 899.05 | 7 |
| 78 | 3 | 3 | 913.14 | 7 |
| 78 | 4 | 3 | 903.62 | 8 |
| 79 | 1 | 1 | 828.38 | 6 |
| 79 | 2 | 1 | 820 | 4 |
| 79 | 3 | 1 | 828.57 | 1 |
| 79 | 4 | 1 | 859.43 | 3 |
| 80 | 1 | 2 | 860.19 | 3 |
| 80 | 2 | 2 | 829.9 | 5 |
| 80 | 3 | 2 | 912.57 | 7 |
| 80 | 4 | 2 | 902.29 | 6 |
| 81 | 1 | 3 | 876.38 | 7 |
| 81 | 2 | 3 | 883.43 | 8 |
| 81 | 3 | 3 | 905.9 | 8 |
| 81 | 4 | 3 | 912.57 | 8 |
| 82 | 1 | 1 | 768.38 | 5 |
| 82 | 2 | 1 | 827.81 | 6 |
| 82 | 3 | 1 | 843.05 | 5 |
| 82 | 4 | 1 | 893.9 | 8 |
| 83 | 1 | 2 | 812.76 | 2 |
| 83 | 2 | 2 | 838.86 | 2 |
| 83 | 3 | 2 | 844.95 | 3 |
| 83 | 4 | 2 | 804.19 | 2 |
| 84 | 1 | 3 | 867.62 | 6 |
| 84 | 2 | 3 | 872.76 | 4 |
| 84 | 3 | 3 | 901.14 | 5 |
| 84 | 4 | 3 | 867.43 | 3 |
| 85 | 1 | 1 | 904 | 7 |
| 85 | 2 | 1 | 908.57 | 8 |
| 85 | 3 | 1 | 908.57 | 6 |
| 85 | 4 | 1 | 908.57 | 6 |
| 86 | 1 | 2 | 780.95 | 6 |
| 86 | 2 | 2 | 808.19 | 5 |
| 86 | 3 | 2 | 736.57 | 6 |
| 86 | 4 | 2 | 821.33 | 5 |
| 87 | 1 | 3 | 889.9 | 8 |
| 87 | 2 | 3 | 901.14 | 8 |
| 87 | 3 | 3 | 908.57 | 8 |
| 87 | 4 | 3 | 908.57 | 8 |
| 88 | 1 | 1 | 736.38 | 2 |
| 88 | 2 | 1 | 766.29 | 4 |
| 88 | 3 | 1 | 884.76 | 5 |
| 88 | 4 | 1 | 880.76 | 7 |
| 89 | 1 | 2 | 828.95 | 5 |
| 89 | 2 | 2 | 859.81 | 5 |
| 89 | 3 | 2 | 880.76 | 6 |
| 89 | 4 | 2 | 901.14 | 7 |
| 90 | 1 | 3 | 800.38 | 8 |
| 90 | 2 | 3 | 860 | 7 |
| 90 | 3 | 3 | 881.14 | 4 |
| 90 | 4 | 3 | 873.9 | 7 |
| 91 | 1 | 1 | 849.33 | 6 |
| 91 | 2 | 1 | 913.14 | 6 |
| 91 | 3 | 1 | 903.24 | 8 |
| 91 | 4 | 1 | 900.38 | 8 |
| 92 | 1 | 2 | 774.1 | 4 |
| 92 | 2 | 2 | 707.81 | 1 |
| 92 | 3 | 2 | 856.95 | 7 |
| 92 | 4 | 2 | 856.95 | 6 |
| 93 | 1 | 3 | 788.38 | 4 |
| 93 | 2 | 3 | 790.67 | 5 |
| 93 | 3 | 3 | 803.81 | 6 |
| 93 | 4 | 3 | 833.33 | 7 |
| 94 | 1 | 1 | 749.52 | 2 |
| 94 | 2 | 1 | 881.9 | 5 |
| 94 | 3 | 1 | 879.62 | 6 |
| 94 | 4 | 1 | 908.57 | 7 |
| 95 | 1 | 2 | 789.14 | 3 |
| 95 | 2 | 2 | 754.67 | 1 |
| 95 | 3 | 2 | 802.86 | 2 |
| 95 | 4 | 2 | 817.52 | 2 |
| 96 | 1 | 3 | 868.76 | 6 |
| 96 | 2 | 3 | 879.24 | 8 |
| 96 | 3 | 3 | 886.86 | 7 |
| 96 | 4 | 3 | 886.1 | 8 |
| 97 | 1 | 1 | 809.52 | 3 |
| 97 | 2 | 1 | 815.62 | 3 |
| 97 | 3 | 1 | 788.19 | 1 |
| 97 | 4 | 1 | 802.48 | 2 |
| 98 | 1 | 2 | 892.57 | 6 |
| 98 | 2 | 2 | 873.9 | 7 |
| 98 | 3 | 2 | 854.48 | 2 |
| 98 | 4 | 2 | 858.86 | 5 |
| 99 | 1 | 3 | 826.1 | 4 |
| 99 | 2 | 3 | 908.57 | 7 |
| 99 | 3 | 3 | 908.57 | 7 |
| 99 | 4 | 3 | 876.57 | 7 |
| 100 | 1 | 1 | 718.1 | 2 |
| 100 | 2 | 1 | 695.43 | 4 |
| 100 | 3 | 1 | 748.95 | 4 |
| 100 | 4 | 1 | 758.48 | 4 |
| 101 | 1 | 2 | 730.48 | 6 |
| 101 | 2 | 2 | 796.95 | 4 |
| 101 | 3 | 2 | 815.24 | 5 |
| 101 | 4 | 2 | 815.24 | 5 |
| 102 | 1 | 3 | 834.29 | 1 |
| 102 | 2 | 3 | 844.38 | 5 |
| 102 | 3 | 3 | 799.24 | 5 |
| 102 | 4 | 3 | 876.95 | 5 |
| 103 | 1 | 1 | 852.19 | 3 |
| 103 | 2 | 1 | 768 | 5 |
| 103 | 3 | 1 | 746.86 | 4 |
| 103 | 4 | 1 | 811.24 | 6 |
| 104 | 1 | 2 | 882.29 | 6 |
| 104 | 2 | 2 | 829.33 | 7 |
| 104 | 3 | 2 | 831.62 | 5 |
| 104 | 4 | 2 | 860.76 | 7 |
| 105 | 1 | 3 | 885.33 | 7 |
| 105 | 2 | 3 | 853.33 | 4 |
| 105 | 3 | 3 | 857.33 | 5 |
| 105 | 4 | 3 | 866.86 | 8 |
| 106 | 1 | 1 | 792.38 | 4 |
| 106 | 2 | 1 | 862.67 | 5 |
| 106 | 3 | 1 | 871.62 | 6 |
| 106 | 4 | 1 | 906.48 | 7 |
| 107 | 1 | 2 | 802.48 | 1 |
| 107 | 2 | 2 | 787.43 | 3 |
| 107 | 3 | 2 | 781.9 | 2 |
| 107 | 4 | 2 | 797.9 | 4 |
| 108 | 1 | 3 | 806.48 | 4 |
| 108 | 2 | 3 | 794.1 | 4 |
| 108 | 3 | 3 | 876.95 | 4 |
| 108 | 4 | 3 | 853.9 | 5 |
| 109 | 1 | 1 | 788 | 5 |
| 109 | 2 | 1 | 836.95 | 4 |
| 109 | 3 | 1 | 749.14 | 4 |
| 109 | 4 | 1 | 782.86 | 5 |
| 110 | 1 | 2 | 815.24 | 5 |
| 110 | 2 | 2 | 819.24 | 7 |
| 110 | 3 | 2 | 852.38 | 5 |
| 110 | 4 | 2 | 828 | 5 |
| 111 | 1 | 3 | 835.62 | 5 |
| 111 | 2 | 3 | 865.52 | 6 |
| 111 | 3 | 3 | 871.43 | 3 |
| 111 | 4 | 3 | 850.86 | 5 |
| 112 | 1 | 1 | 869.71 | 7 |
| 112 | 2 | 1 | 908.57 | 7 |
| 112 | 3 | 1 | 825.33 | 8 |
| 112 | 4 | 1 | 908.57 | 8 |
| 113 | 1 | 2 | 832 | 3 |
| 113 | 2 | 2 | 839.43 | 7 |
| 113 | 3 | 2 | 848 | 3 |
| 113 | 4 | 2 | 840.57 | 3 |
| 114 | 1 | 3 | 802.86 | 3 |
| 114 | 2 | 3 | 817.9 | 7 |
| 114 | 3 | 3 | 855.43 | 7 |
| 114 | 4 | 3 | 869.71 | 4 |
| 115 | 1 | 1 | 862.1 | 6 |
| 115 | 2 | 1 | 795.81 | 4 |
| 115 | 3 | 1 | 889.71 | 7 |
| 115 | 4 | 1 | 908.57 | 6 |
| 116 | 1 | 2 | 768.95 | 1 |
| 116 | 2 | 2 | 815.24 | 2 |
| 116 | 3 | 2 | 813.14 | 3 |
| 116 | 4 | 2 | 754.1 | 3 |
| 117 | 1 | 3 | 881.9 | 6 |
| 117 | 2 | 3 | 863.24 | 7 |
| 117 | 3 | 3 | 872.76 | 7 |
| 117 | 4 | 3 | 898.86 | 6 |
